# Microbial defenses against mobile genetic elements and viruses: Who defends whom from what?

**DOI:** 10.1371/journal.pbio.3001514

**Published:** 2022-01-13

**Authors:** Eduardo P. C. Rocha, David Bikard

**Affiliations:** 1 Institut Pasteur, Université de Paris, CNRS UMR3525, Microbial Evolutionary Genomics, Paris, France; 2 Institut Pasteur, Université de Paris, Synthetic Biology, Department of Microbiology, Paris, France

## Abstract

Prokaryotes have numerous mobile genetic elements (MGEs) that mediate horizontal gene transfer (HGT) between cells. These elements can be costly, even deadly, and cells use numerous defense systems to filter, control, or inactivate them. Recent studies have shown that prophages, conjugative elements, their parasites (phage satellites and mobilizable elements), and other poorly described MGEs encode defense systems homologous to those of bacteria. These constitute a significant fraction of the repertoire of cellular defense genes. As components of MGEs, these defense systems have presumably evolved to provide them, not the cell, adaptive functions. While the interests of the host and MGEs are aligned when they face a common threat such as an infection by a virulent phage, defensive functions carried by MGEs might also play more selfish roles to fend off other antagonistic MGEs or to ensure their maintenance in the cell. MGEs are eventually lost from the surviving host genomes by mutational processes and their defense systems can be co-opted when they provide an advantage to the cell. The abundance of defense systems in MGEs thus sheds new light on the role, effect, and fate of the so-called “cellular defense systems,” whereby they are not only merely microbial defensive weapons in a 2-partner arms race, but also tools of intragenomic conflict between multiple genetic elements with divergent interests that shape cell fate and gene flow at the population level.

## Abstract 2

Recent data suggests that many defence systems found in the genomes of Bacteria and Archaea are encoded in mobile genetic elements. This Unsolved Mystery maintains that this realization challenges our understanding of their role in cells and raises key novel questions: Who is defending? From what? And what is the role of the cellular genome in this complex network of antagonistic interactions between mobile genetic elements?

## Introduction: Mobile genetic elements drive gene flow at a (sometimes hefty) cost

Horizontal gene transfer (HGT) allows bacteria and archaea to rapidly match novel ecological challenges and opportunities. HGT is most frequently mediated by mobile genetic elements (MGEs) like bacteriophages (phages) and conjugative elements that are present in most genomes, often in multiple copies (for definitions, see Box 1). These elements can autonomously transfer themselves from one cell to another using viral particles or conjugative pili, processes that also contribute to the exchange of chromosomal DNA. Besides their ability to drive HGT, many MGEs encode traits adaptive to the host cell. For example, key virulence factors in many human pathogens are encoded in prophages, and the transfer of antibiotic resistance genes is driven by conjugative elements [1,2]. By increasing the host fitness in specific contexts, these traits contribute to the proliferation of MGEs in communities, i.e., they directly contribute to MGE fitness. In other contexts, these traits can be costly and, together with the costs of vertical and horizontal transfer, lower the host fitness [3]. As a result of these costs, MGEs rarely remain in genomes for long periods of time [4], and different strains tend to carry very different repertoires of prophages, plasmids, or transposable elements [5–8].

**Box 1. Glossary.**

**Abortive infection**: process by which an infected cell induces its own death before the phage can complete its replication cycle. This strategy is employed by a large diversity of defense systems that sense infection and trigger cell death through various mechanisms.

**Allelic recombination**: a process, usually using homologous recombination, which results in allelic exchanges between very similar genes present in different genomes. One uses the term recombination for exchanges leading to transfer of polymorphism in conserved genes (gene conversion) from one bacterium to another and HGT for transfer of novel genes.

**Balancing selection**: evolutionary process where there is selection to maintain polymorphism in the population. For example, it can favor the diversity of alleles in genomes (e.g., multiple polymorphic copies of a gene family in a genome), the preservation of rare alleles in populations (negative frequency–dependent selection selection), or of different alleles in different environments or different moments.

**Chromosome hotspots**: regions of bacterial chromosomes with high turnover of genetic material, i.e., with frequent insertion and deletion of genes acquired by HGT (often in MGEs).

**Conjugative elements**: plasmids or integrative conjugative elements (ICEs) can produce a mating pair formation structure at the cell envelope to transfer DNA between neighboring cells.

**Clustered regularly interspaced short palindromic repeats/CRISPR associated protein (CRISPR/Cas) systems**: provide adaptive immunity based on spacer sequences within CRISPR arrays. These sequences guide nucleases to destroy cognate invading genetic elements.

**Defense islands**: chromosomal loci with high density of defense systems.

**Homologous recombination**: molecular process that results in the joining of two DNA molecules at a region of homology. Can result in the exchange of polymorphism between the chromosome and exogenous DNA sequences.

**Horizontal gene transfer (HGT)**: transfer of novel genes from one microbial cell to another.

**Mobile genetic elements (MGEs)**: semiautonomous genetic elements that are capable of mobility within or between genomes. They include phages and conjugative elements capable of autonomous transfer between cells, satellites and mobilizable elements capable of hijacking the former ones, and many other elements whose means of horizontal transfer are unknown.

**Mobilizable elements**: plasmids or integrative mobilizable elements (IMEs) capable of hijacking mating pore formation structures of conjugative elements for their own transfer.

**Phages**: bacterial viruses.

**Phage satellites**: MGEs capable of hijacking phage particles produced by phages for their own transfer.

**Restriction–modification (R–M) systems**: use specific methylation patterns to distinguish self from nonself. Incoming MGEs lacking compatible methylation patterns are degraded by a nuclease.

**Retron**: genomic elements encoding a reverse transcriptase and a noncoding RNA that is partly reverse transcribed to form a RNA–DNA hybrid. Some retrons have been recently shown to be antiphage defense systems.

**Transduction**: processes by which phage particles transfer bacterial DNA between cells.

Conjugative elements and phages also have molecular parasites that use their mechanisms of horizontal transmission to transfer between cells. For example, viral particles produced by phages can be hijacked by phage satellites [9], and conjugative pili can be used by so-called mobilizable elements [10]. Many other MGEs lack known mechanisms of horizontal transmission and may transfer between cells by exploiting phages and conjugative elements [10]. It must be noted that the presence of a MGE affects the frequency of other MGEs in the cell. This is the case of the mobility of multiple conjugative plasmids in cells [11], of the abovementioned mobilizable plasmids and phage satellites that cotransfer in synchrony with conjugative elements and phages, and of phages that use the conjugative pilus as a receptor for cell infection [12]. Finally, infection by a MGE may spur the transfer of others. Phage infection favors the transfer of SXT-like integrative conjugative elements (ICEs) [13] and conjugation-induced SOS response activates MGEs in the recipient cells [14]. Transposable elements are a particularly important family of mobile elements that is very abundant within other MGEs and facilitates genetic exchanges between them and with the host [15]. The cellular genome thus harbors a large diversity of MGEs establishing complex interactions among each other and with the host cell.

The associations between the host and its MGEs lay on a continuum going from pure parasitism to intimate mutualism because vertical and horizontal transmission of MGEs impose fitness costs to the cell that may eventually be outweighed by advantageous traits encoded by them. Virulent phages are at the edge of maximal virulence in this continuum since their successful infection implicates cell death. The fitness effects of the remaining MGEs, and of the accessory traits they encode, are more diverse and vary with the physiological state of the cell and the presence of competing MGEs. Temperate phages are striking examples of such ambiguity. Their integration in the genome can provide novel adaptive traits [16], but their subsequent excision from the genome usually ends in host death [17]. The richness of the interactions between MGEs and the host make their impact contingent on a specific cellular context, i.e., this impact varies with the strain genetic background. MGEs that are parasites of other MGEs impact the fitness of the latter. If this impact is very high and the parasitized MGE is deleterious to bacteria, then the parasite of the parasite benefits the host cell. For example, some satellites can abolish phage transmission by release of viral particles exclusively packaged with the satellite genome [18]. Although this process still ends in cell death, the inhibitory effect of the satellite on phage reproduction blocks its epidemic growth, thereby protecting the microbial population. Since most genomes contain plasmids, prophages, and other MGEs [10,19], and virulent phages are extremely abundant in the environment [20], the fate of cells often hangs in the outcome of their interaction with MGEs and that of MGEs among themselves.

**Fig 1:**
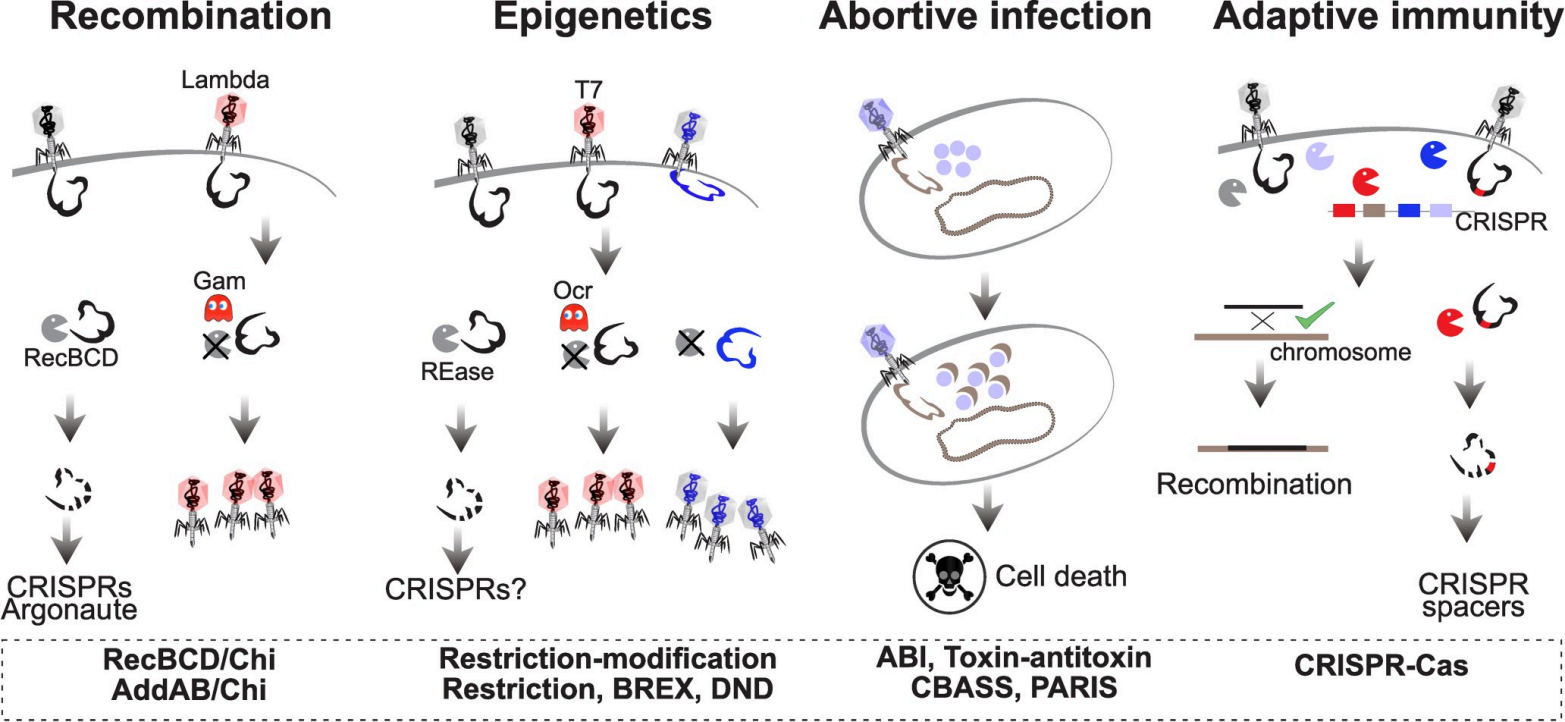
Examples of defense systems discussed in the text. Homologous **recombination** via the RecBCD pathway can defend from MGEs because RecBCD is a powerful exonuclease that degrades linear double-stranded DNA (but is inactivated by some phage encoded proteins, like Gam). **Epigenetic** modifications are the basis of many defense systems that can be counteracted by phage proteins, like Ocr in phage T7, or by epigenetic modification of phage DNA. **Abortive infection** is a costly defense strategy that induces cell growth arrest or death when a threat to the cell is detected. **Adaptive immunity** by CRISPR/Cas systems targets MGEs with specific DNA sequences, while allowing other exogenous DNA to remain in the cell and eventually recombine with the chromosome. CRISPR/Cas, clustered regularly interspaced short palindromic repeats/CRISPR associated protein; MGE, mobile genetic element.

The potential for conflicts in the interactions between MGEs and the host led to the evolution of defense mechanisms to filter, control, or inactivate them [21,22]. These systems are now being unraveled at fast pace even though their mechanisms are in many cases still poorly understood [23,24]. The extensive description of these systems falls outside the scope of this text and can be found elsewhere [25–28]. Some defenses are part of core cellular systems and provide protection from MGEs as part of a broader set of cellular functions (Fig 1). For example, RecBCD is a powerful exonuclease involved in the repair of double-strand breaks by homologous recombination. It degrades linear double-stranded DNA until it meets a Chi site beyond which it loads the recombinase RecA. Phages lacking Chi sites are rapidly degraded by the enzyme [29]. The resulting DNA can be recovered by other defense systems, like clustered regularly interspaced short palindromic repeats/CRISPR associated protein (CRISPR/Cas) or the Argonaut, to build more specific defenses against subsequent infections by the same phages [30,31]. Hence, generic defense systems that are part of the host core genome may nourish more specialized systems for more specific protection. Interestingly, Lambdoid phages in *Escherichia coli* either lack Chi sites, in which case they usually encode anti-RecBCD systems to block RecBCD and produce their own recombination systems, or have many Chi sites and use the host homologous recombination machinery for their own replication [32]. Bacteria have evolved responses to protect themselves from phage-encoded anti-RecBCD systems. A retron was recently discovered that induces cell death when the RecBCD function is compromised, i.e., the retron is a guardian of RecBCD and has a protective anti-anti-RecBCD function [33].

Contrary to RecBCD, many defense systems are not involved in core cellular processes. Instead, they are specialized in providing innate or adaptive immunity. Restriction–modification (R–M) systems, by far the most abundant defense systems in bacterial genomes [34,35], provide excellent illustrations of the evolutionary processes resulting in the evolution of defense and counter-defense systems (Fig 1). They imprint epigenetically the cellular genome and inactivate (restrict) infecting MGEs lacking the adequate DNA modifications. As a response, some phages counteract the activity of R–M systems by either producing anti-restriction proteins or by extensively modifying their DNA [36,37]. Anti-restriction functions can, in turn, be recognized by bacterial anti-anti-restriction systems that provide a second layer of resistance when R–M fails, e.g., by inducing cell death (abortive infection, Fig 1) [38]. As a complement, phages with extensive modifications in their DNA can be recognized by specific bacterial antimethylation restriction systems [39,40]. The evolution of this tit-for-tat mechanisms can go very far. For example, phage T4 encodes an anti-restriction system that can be recognized by hosts encoding an anti-anti-restriction system that induce cell death by tRNA cleavage. This abortive infection system can be canceled by T4 because it can repair the cleaved tRNAs using a pair of proteins that constitute an anti-anti-anti-restriction system [41].

**Fig 2:**
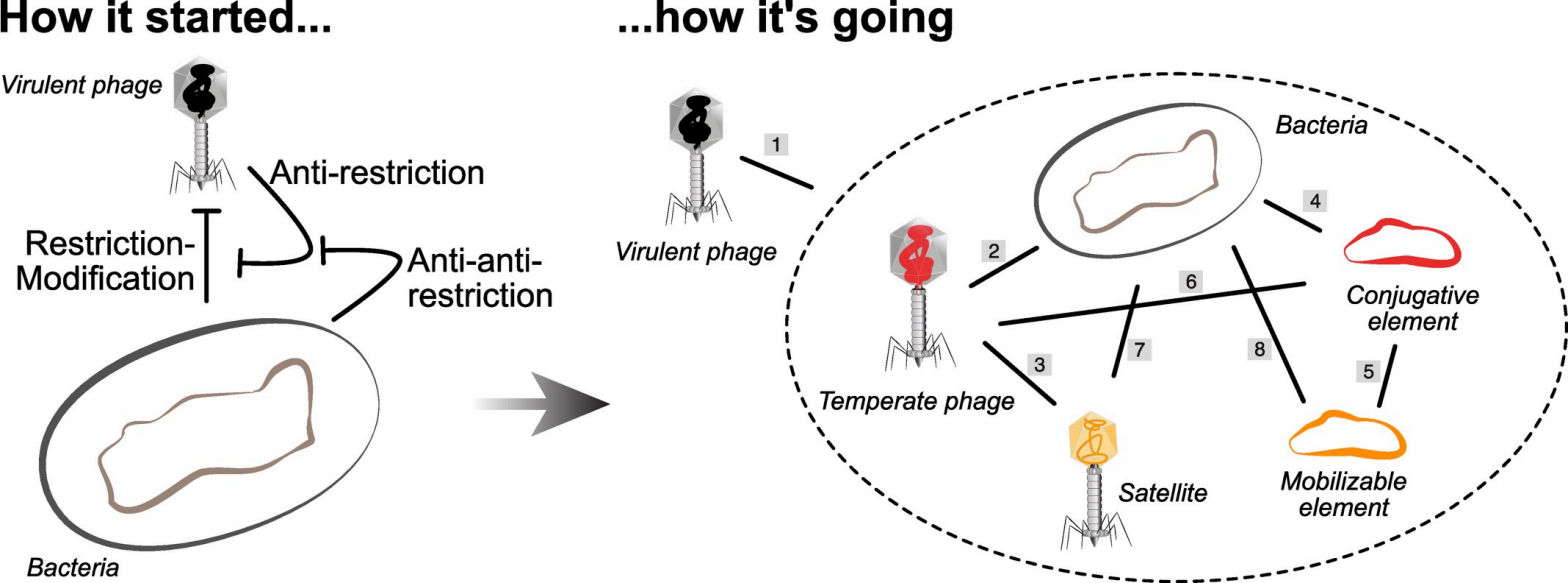
Defense and antidefense systems are often studied in the context of the interaction between one host and one MGE, usually a virulent phage (left). Yet, the presence of numerous MGEs in populations and their ability to encode their own defense systems renders the picture more complex (right). Virulent phages establish antagonistic interactions with the other MGEs and the cell (1). But the associations between the other MGEs and the cell can be more diverse (2 to 7). Temperate phages and conjugative plasmids exploit their cellular host (2 and 4) and can be exploited by other MGEs (3 and 5). Plasmids often encode systems that are effective barriers to phages, e.g., R–M (6). Phages are a threat to plasmids when they kill the host cell (6). Satellites may benefit the host by diminishing phage infection (7). Most of these interactions (2 to 8) can at times be beneficial to both partners, e.g., when a conjugative plasmid provides a nosocomial bacterium with antibiotic resistance. MGE, mobile genetic element.

Defense and counter-defense systems are often studied in the light of the antagonistic interaction between one host and a virulent phage. But the reality is much more complex and interesting because many of the systems found in bacterial genomes and once thought to be dedicated to the defense of the cell are actually encoded in MGEs. This includes systems encoded in temperate phages [8,42–45], satellites [38,46,47], conjugative elements [13,48–50], mobilizable plasmids [34], and genomic islands acquired by HGT [8,44,51]. For example, defense systems in *Vibrio* and in other species are a significant part of the species’ accessory genome (12% to 20%) and are very frequently encoded in MGEs, many of which are poorly known [8]. It is important to note that the presence of occasional defense systems in phages or plasmids has been known for decades. What these recent observations highlight is that a large fraction of the so-called cell defense systems are encoded in MGEs. This raises intriguing questions concerning the role, function, and evolution of the so-called cellular defense systems (Fig 2).

### Why are there so many defense systems in each genome?

Most bacterial genomes encode several R–Ms, but often also CRISPR/Cas, retrons, and many other defense systems [35]. For example, the first 2 sequenced genomes of *Helicobacter pylori* encode a total of more than 20 putative R–M systems [52], and genomes with multiple CRISPR arrays and Cas systems are frequent [53]. The fast pace of discovery of novel defense and counter-defense systems suggests that they may account for a significant number of the unknown function genes in genomes. The numerous defense systems of some genomes protect the cell from a broad range of MGEs, counteracting the latter’s tendency to evolve counter-defenses. It could also facilitate building up multiple layers of cell defenses that may culminate in cell death when all else fails [27]. In this view, cell defense systems are numerous because they make a multilayered immune system to tackle different elements. Yet, defense systems can be costly [54], because of production costs when they are required at high concentration [55], because their activity can be energetically costly [56], or because they may be incompatible with other cellular mechanisms [57]. They can also kill the cell by autoimmunity [58]. Hence, the number of systems under selection for defense by the host cell is expected to depend on the balance between these costs and the rewards given by their ability to protect hosts from MGEs.

The observations that genomes have many MGEs and that these encode many defense systems provide an alternative or complementary explanation for why genomes contain so many such systems. Genomes contain many defense systems because they are acquired within the multiple MGEs that infect microbial cells. Since there are many MGEs in a cell, these sum up to a considerable number of defense genes. Such MGE-encoded defenses may also be multilayered. For example, *E*. *coli* plasmids encoding both BREX and type IV restriction systems have recently been shown to provide complementary protection from phages [59]. This does not exclude the possibility that cells select for multiple systems of defense, but does suggest that to understand their frequency in cells one must also account for the infectivity of MGEs. This means that the multiplicity of systems in cellular genomes might be a consequence of the high transmissibility and abundance of MGEs, not (only) the result of natural selection for protection of the cell. It is therefore possible that cells encode more defense systems than the theoretical optimal number expected for a host cell, simply because many of the systems are selected for their presence in the MGE, not in the host.

### Why are defense systems very diverse within species?

Defense systems tend to be different across strains of a species [21,51] and are a significant part of the genetic differences between closely related strains of *Vibrio* spp. [8]. Why are defense systems so different among strains of a species? The coevolutionary dynamics between defenses and counter-defenses contributes to an endless process of genetic diversification that is often understood in the context of balancing selection [60]. These are processes where natural selection favors the existence of genetic polymorphism. Interestingly, balancing selection resulting in the presence of diverse defense systems in populations is observed in many immune systems, from bacteria to humans [61].

Balancing selection can occur by multiple mechanisms. First, it is harder for a parasite to spread in a population with diverse host defenses even in simple systems [62]. For example, genetic diversity in the host CRISPR/Cas spacers that target a phage favor the host and make the mutational escape by the phage more difficult [63]. The presence of various systems providing immunity from MGEs within microbial populations increases the likelihood that some individuals are protected, in what has been described as distributed (pan) immunity [26]. Relative to microbial genomes, MGEs are more constrained in the number of genes they can carry, especially those packaged in viral particles. Yet, some also carry multiple defense systems [8,13,38,44], which may allow them to infect different hosts or fend off different MGEs.

Second, variations in time and space of the density, type, and behavior of MGEs may favor different cellular defense systems in different situations. The distribution of MGEs varies across bacterial habitats [64] and across environmental conditions within habitats [65]. Hence, locally adapted microbial populations may select for different systems that tackle different types of MGEs resulting in variable defense repertoires across a bacterial species. This is also applicable to defense systems encoded in MGEs. Their defense systems can be under balancing selection because the hosts and MGEs they encounter vary in space and time.

Third, clones that are more abundant in a habitat are more susceptible to phages, because of their density [66]. In this context, negative frequency–dependent selection may result in selection of rare alleles [67], i.e., bacterial clones with defense systems that are rare in the population. As the population of individuals with the rare adaptive defense increases, antagonists with the ability to infect it also rise in frequency because they have more hosts available. This decreases the advantage of the initial clone and eventually cancels it when novel rare clones resistant to the MGEs emerge, thereby restarting the process of negative frequency–dependent selection. While negative frequency dependence in host–pathogen interactions has been extensively studied [61], there is a paucity of data on its role in MGE–host interactions.

### How is immunity gained?

What are the molecular mechanisms driving the variation of bacterial defenses? Some systems have dedicated molecular mechanisms for their own variation. For example, CRISPR/Cas systems drive their own diversification by the acquisition of spacers to target novel elements [22]. Some R–M systems can also rapidly change their sequence specificity through recombination [68]. These mechanisms allow the host to fine-tune its defenses either directly to specific MGEs (CRISPR/Cas systems) or in more generic ways (variation in restriction sites).

Yet, the available evidence suggests that HGT and gene loss have major complementary roles in the diversification of defense repertoires at the species level. Accordingly, not only there is extensive evidence of the HGT of defense systems in MGEs [69], but also their pseudogenes have been observed for many R–M [52] and CRISPR/Cas systems [70]. The abundance of defense systems in MGEs suggests a very straightforward mechanism for the acquisitions of defense systems by the host: Systems are transferred across strains by the MGEs encoding them. Furthermore, MGEs are gained at high rates because of their infectiousness (explaining acquisition), and they are frequently lost from populations because of their cost (explaining loss). The rates of gain and loss of defense systems may thus be partly caused by the mobility and lability of the mobile elements encoding them.

Beyond explaining the acquisition of novel systems, the presence of defense systems in MGEs also offers some clues on how entirely novel defense strategies emerge. The recent discovery of many antiphage systems shows that they frequently consist in an assemblage of protein domains that are also present in proteins implicated in other cellular processes such as nucleases, kinases, deaminases, proteases, or ATPases [71]. For instance, the Stk2 defense kinase is part of a family of kinases whose members are implicated in various cellular process such as the control of the cell cycle or the exit of dormancy [72]. The antiphage viperins are close homologues to GTP cyclases involved in other functions [73]. The co-option of proteins, or protein domains, with other functions, and the creation of novel assemblages leading to genetic innovation by recombination and mutation are likely facilitated by the horizontal transfer of defense systems across genetic backgrounds [74]. While successful functional innovations by co-option of these systems may be unlikely, the very frequent transfer of systems and their rapid evolution may result in such a high rate of novel combinations of domains that some will eventually evolve to become novel defense systems. Such processes of co-option may have been at the independent origins of both Cas-9 and Cas-12 proteins from transposon-encoded RNA-guided endonucleases [75,76].

Novel defense systems, even if initially not part of MGEs, will eventually be captured by MGEs for their own use, with the consequence that they will be spread across microbial lineages. Transposases may play key roles in the process of translocating these systems from the chromosome to MGEs and vice versa. The subsequent transfer of defense systems to different genetic backgrounds is expected to favor the spread of defense systems that are robust to such changes. Accordingly, there is a broad distribution of most defense systems across the bacterial kingdom [35]. It is also interesting to note the surprisingly broad activity of some defense systems recently described [23]. Cloning these genetic systems from distant species into *E*. *coli* and *Bacillus subtilis* yields defense phenotypes. The presence of defense systems on MGE that move across species might thus favor broad defense capabilities and mechanisms tolerant to changes in the genetic background.

### Defending whom from what?

The rapid pace of discovery of novel defense systems has been facilitated by the use of assays where cells are challenged by virulent phages. As a result, the role of defense systems tends to be discussed in the light of phage–bacteria interactions. It does seem reasonable to assume that systems present in a microbial genome for a long time are protecting it from MGEs and especially against virulent phages given their lethality for the cell. Yet, systems encoded in MGEs are more likely to be selected because they benefit the MGE. In certain cases, a system increases the fitness of both MGE and host. For example, defense systems encoded in P4-like satellites were shown experimentally to protect the cell from several phages that the P4 element cannot exploit [38]. In this case, the satellite and the cell have the same interest in preventing infection by phages that can kill the cell. In general, both MGEs and hosts will gain from preventing infection by virulent phages, explaining why MGEs defenses seem to target them frequently.

The interests of the MGE and the cell may not be so well aligned in other circumstances. In some cases, the advantage of the MGE defense system to the cell may be transient. Temperate phages that defend the cells from virulent phages are common [8,43,77] and provide a temporary relief to the host. But they may have little long-term impact in bacterial fitness if the victorious temperate phage is induced and lyses the cell. This is also exemplified by the exclusion systems encoded by conjugative systems or phages to fend off closely related elements [78,79]. Historically, these mechanisms have not been included in defense systems, but they are costly mechanisms that protect the cell from infection by MGEs, i.e., they fit the definition of defense systems as applied here to many other MGE genes. For example, the surface exclusion system of plasmid F prevents infection by similar plasmids thanks to the production of thousands of copies of an outer membrane protein that accounts for a large part of the plasmid carrier cost [80]. A plasmid or a phage encoding an expensive exclusion/defense system against a similar element is engaging in an antagonistic interaction whose cost to the cell is large whereas the reward may be small because one such element is already in the cell (even if exclusion may protect cell integrity from too many simultaneous infections [79]). An even more extreme case concerns phages encoding defense or antidefense systems against their satellites. These are engaging in an interaction with their parasites in a way that resembles their own interaction with the cell (but with their own position reversed as they are now the ones being exploited) [47]. Such phage-encoded defense systems could be highly deleterious to the cell because they remove a protective satellite and favor a phage that will eventually kill the host.

The misalignment of interests between MGEs and the host is particularly striking when it concerns abortive infection systems, because these are extremely costly to the cell [27]. The traditional view is that such strategies can only be selected in very particular cases favoring cooperation between individuals, e.g., in structured environments where the death of a cell benefits its neighboring kin [81]. A recent investigation of abortive infection provided by retron elements suggests that retron-encoding bacteria lose in competition with bacteria lacking the retron when challenged by a phage even in a structured environment [82]. Yet, genomic data suggest that abortive infection systems are very frequent [35], which requires an explanation. The presence of abortive infection systems on MGEs could facilitate the control of epidemics of competitive elements and would justify their abundance in the host. Such systems could be deleterious to the host if they drive cell death upon infection by elements with little negative impact on its fitness. But in other circumstances, the presence of these systems in MGEs could benefit the host by enforcing cooperation [83], since the transfer of the MGEs to sensitive hosts spreads the abortive system and therefore favors the cooperative process.

To understand the fitness impact of defense systems, it is thus important to know if they are encoded in MGEs. The identification of functional MGEs is difficult both computationally and experimentally, since many MGEs are poorly known and many of the others are defective [74]. It is often even more difficult to predict which genetic elements are being targeted by the defense system. That many systems are effective against virulent phages may be in part the result of ascertainment biases, since virulent phages are often used to identify defense systems. One might also argue that virulent phages are going to be targeted by hosts and most MGEs because they kill the host and its MGEs. However, many systems, among which all those using epigenetic markers like R–M, target generic exogenous DNA independently of it being part of a phage genome. This makes it particularly hard to know who they were selected to target.

CRISPR/Cas systems are unique among defense systems because they keep a record of the elements against which they defend and therefore allow to pinpoint the elements that have been sufficiently deleterious to result in selection of the corresponding CRISPR spacers. The analysis of the spacer content can thus inform on the selection pressure that maintain CRISPR immunity. While some CRISPR/Cas systems predominantly carry spacers targeting bacteriophages, others like type IV CRISPR/Cas systems predominantly carry spacers against plasmids [49], and many carry both [84]. These results suggest that systems encoded in MGEs may be targeting other competing MGE that are not costly to the cell. They may even be targeting elements that are adaptive to the cell or targeting the cell itself (e.g., addictive systems or antidefense systems), thereby lowering bacterial fitness. Knowing which genetic elements are being targeted in nature will require a better mechanistic understanding of the defense systems and the ecological contexts where they are selected for.

### How do defense systems affect gene flow?

**Fig 3:**
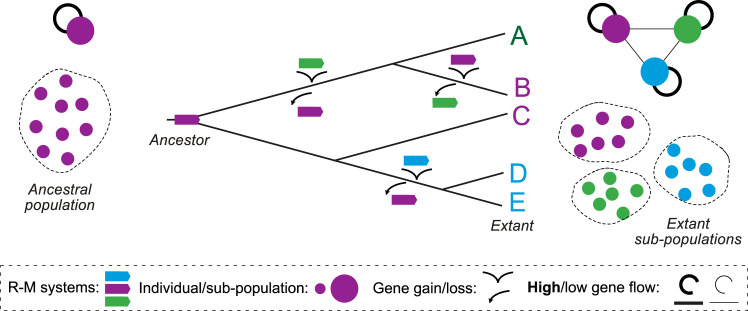
Diversification of R–M systems changes gene flow within species. When a population has a single R–M system (left), HGT between cells is not affected by restriction. As the diversity of systems increases (phylogenetic tree at the center), the subpopulations of individuals with similar R–M systems exchange genes at higher rates (high flow) than those with different R–M systems (low gene flow, right top), leading to fragmentation of gene flow in populations (right bottom). HGT, horizontal gene transfer; R–M, restriction–modification.

Acquisition of defense systems requires HGT, but defense systems are expected to decrease the rates of transfer of MGEs, and thus decrease HGT. Gene flow, including allelic recombination and acquisition of novel genes by HGT, is a key driver of bacterial evolution, and there is an evolutionary cost to restricting it. For example, epidemic *Vibrio cholerae* strains depend on a prophage for a key virulence factor (the cholera toxin). When they are infected by SXT-like conjugative elements carrying defense systems, they are hampered in their ability to acquire the toxin [13]. More generally, a computational analysis of approximately 80 species showed that gene flow is decreased between strains with incompatible R–M systems [85]. When R–M systems diversify within populations, because of bacterial/MGE antagonistic coevolution, DNA exchanges become more frequent between strains with similar systems (Fig 3). As a result, defense systems have the potential to fragment gene flow within bacterial populations.

The presence of mechanisms of defense may impact gene flow in diverse ways. One would expect that very generic systems, like R–M, would have an important effect on restricting gene flow, whereas more targeted systems, like CRISPR/Cas, would tend to affect gene flow driven by a few particularly deleterious elements. Accordingly, the impact of CRISPR/Cas in restricting gene flow has remained controversial [86,87].

The negative impact of defense systems on gene flow has been regarded as a costly by-product of selection for protection of the cell. But MGE defense systems may be selected exactly because they block HGT to prevent the cell from acquiring competitor MGEs. The resulting sexual barriers are advantageous for the MGE but can be deleterious to the cell. Yet, these barriers are not unbreakable. The presence of multiple MGEs in genomes is in itself an indication of this. Accordingly, R–M systems only provide transient protection from phages [88], because one single successful infection is enough to result in correctly methylated phages that can pass the restriction barrier and then propagate across the population. Further work is needed to quantify the impact of different defense systems in gene flow, to identify the types of MGEs that are most affected, and to understand how defenses affect host evolvability.

The effect of defense systems on gene flow is not always negative. Transduction, the transfer of bacterial DNA in viral particles, is favored by the existence of CRISPR/Cas systems in recipient cells when they target the phage DNA (Fig 1). This is because the CRISPR/Cas system targets the phage DNA, protecting the cell, but not the bacterial DNA coming within viral particles, resulting in cells that receive exogenous cellular DNA while being protected from phages [89]. In this case, the defense system facilitates gene flow.

### Is it defense, counter-defense, addiction, or something else?

While many systems have been called defensive relative to their ability to defend bacteria or MGEs from other MGEs, they may be addictive or attack systems when part of MGEs. A striking example is provided by phage–satellite interactions. The reproduction of virulent phages of the ICP1 family in *V*. *cholerae* is abolished by phage-inducible chromosomal island-like elements (PLEs) [18]. In response, ICP1 phages have evolved the ability to encode a CRISPR/Cas system or specific nucleases that eliminate the satellite [90]. In this context, they could be regarded as attack systems from the point of view of the bacterium, because their success results in cell death. They could also be regarded as phage counter-defenses, if satellites are considered as a bacterial defense system. There is thus some ambiguity between functions of defense, counter-defense, and attack, depending on the perspective of the observer.

Some systems may have multiple roles specifically when encoded in MGEs. R–M systems contribute to the stabilization of plasmids in the cell by acting as poison–antidote addictive systems [91]. In such cases, loss of the plasmid and its R–M system prevents further expression of the latter. Since endonucleases have longer half-lives than methylases, this eventually results in genomes that are restricted because they are insufficiently methylated. R–Ms are thus part of the attack arsenal of plasmids. Yet, these R–M systems can also protect the consortium (cell and plasmid) from infection by other MGEs, thereby acting as cell defense systems. Plasmids also frequently encode toxin–antitoxin systems that behave as addiction systems [92], some of which are implicated in phage defense. Homologues of cell defense systems encoded in MGEs can thus be addiction tools with positive side effects in cellular defense. It is possible that such systems have started as genetic elements that propagate selfishly in genomes because of their addictive properties and have later been co-opted to become defense systems (although the inverse scenario cannot be excluded at this stage).

### Are MGEs at the origin of “defense islands”?

It was observed a decade ago that defense systems are often clustered in a few loci in microbial chromosomes [93]. This characteristic was leveraged into a systematic method to discover novel systems by colocalization with known ones [23]. Interestingly, recent data have revealed that antidefense systems, both anti-R–M and anti-CRISPR/Cas, also tend to cluster in bacterial genomes, often in recognizable MGEs [94]. The clustering of these systems could result from selection for the coregulation of their expression, but there is very little evidence of that.

**Fig 4:**
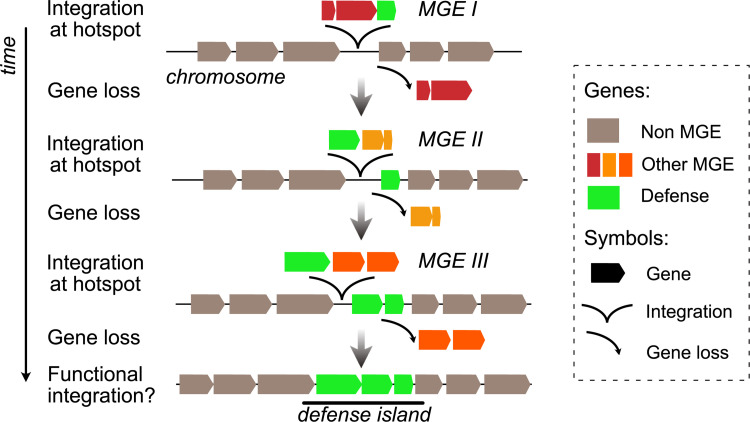
MGE turnover at hotspots may result in defense islands. MGEs tend to integrate the chromosome at a few hotspots and may subsequently be inactivated by mutations resulting in the loss of genes that are not adaptive to the host. The succession of integration/partial deletion of MGEs if accompanied by the co-option of the defense systems may result in clusters (or islands) of defense systems in hotspots. MGE, mobile genetic element.

The presence of defense systems in MGEs provides a simple explanation for the colocalization of defense and counter-defense systems in a few locations of the bacterial chromosome (Fig 4). Genes acquired by HGT, and MGEs in particular, tend to integrate at a small number of chromosome hotspots [95–98], and some of these were found to have defense systems over a decade ago [51]. These MGEs may degenerate by the accumulation of mutations, deletions, and insertions. Chromosome hotspots are thus littered with remnants of previous events of transfer. As MGEs are integrated and eventually degrade in the hotspot, some genes may remain functional because they are adaptive for the cell [74]. Since MGEs often carry defense and antidefense systems, their rapid turnover in hotspots may be accompanied by selection for the conservation of some of their defense systems. Ultimately, this could result in their co-option by the host cell. As rounds of MGE integration/degradation succeed in natural history, the remnant co-opted defense systems form clusters in the chromosome, i.e., they form defense islands. Since studies on defense islands have not focused on separating those in MGEs from the others, it remains unclear whether defense islands might simply be an assemblage of functional MGEs or whether integration/degradation events play a significant role in stabilizing clusters of defense systems.

The clustering of defense systems may facilitate the evolution of functional interactions between them or the coregulation of their expression. For example, it has been observed that type I and type III CRISPR/Cas systems provide 2 integrated levels of defense against phages [99]. These systems are often colocalized [53]. Even if advantages of their colocalization in the genome are yet unclear, it may facilitate cotranscription or coevolution of the systems. The clustering of these systems in islands could also facilitate their subsequent transfer as a block by HGT to other cells. This could occur by mechanisms able to transfer large genetic loci such as conjugation starting a conjugative element integrated in the chromosome or lateral transduction starting from a neighboring phage [47].

### Outlook

MGEs of bacteria and archaea encode accessory functions of adaptive value for the host. That many of these accessory functions concern systems facilitating host infection or MGE protection from other elements testifies to the importance of such interactions for the fitness of MGEs. These defense systems may also be adaptive to the host, but this should not be taken for granted. Having this conceptual framework in mind can aid the field move forward along the following lines.

- Identify novel defense systems. Many defense systems are poorly known and probably many more remain to be uncovered. The recent expansion in the number and type of defense systems occurred because researchers searched for novel systems colocalizing with previously known ones. Many novel systems may be awaiting discovery among the countless MGEs present across microbial genomes. Since these genes are often found at specific locations in MGEs, e.g., near the *cos* site of P4-like satellites [38], these locations may be treasure troves of novel defense systems.
- Understand the mechanisms of defense. Most of the studies on the mechanisms of defense systems use virulent phages as targets. Yet, systems encoded by MGEs may target different elements and having this information may result in the discovery of novel molecular mechanisms, especially among systems targeting specific MGE functions. Recent works have revealed defense systems targeting specific molecular mechanisms of phages [73,100]. Maybe other defense systems target mechanisms of conjugative elements or other MGEs.
- Identify counter-defense mechanisms. Counter-defense mechanisms are now being identified for the best-known mechanisms of defense. Integrating the knowledge of the existence of mechanism of defense in an element, its molecular mechanism, and the elements being targeted could provide important clues on where to find novel antidefense systems from known or novel defense systems.
- Inference of the networks of defenses. As defense systems provide multiple layers of defense against MGEs, it is important to understand what these layers are and how they interact. Ultimately, immune systems of bacteria might rely on complex networks of functional and genetic interactions between defense systems that provide a robust and thorough response to most parasites. These networks may resemble those of the eukaryotic immune system. The parallel between innate (R–M) and adaptive (CRISPR/Cas) immune systems has been drawn before [101], and some defense systems have homologues in eukaryotes and bacteria [73].
- Identification of the mechanisms generating novel defense systems. These evolutionary mechanisms may also share similarities across the tree of life, since some regulatory elements or components of the immune system of vertebrates and plants also derive from co-options of MGEs [102, 103]. Balancing selection seems to explain the evolutionary patterns of defense systems in bacteria, plants and animals [60]. Yet, one must keep in mind that a lot of the variation in the bacterial immune response is associated with rapid gain and loss of defense systems, many of which in MGEs, which is different from the processes driving the diversification of immune systems of vertebrates.
- Mapping the interactions between MGEs and the host. Knowing the mechanisms of defense systems carried by a specific MGE can hint at their possible targets and therefore reveal the MGEs (or host) affected by the element. This can be leveraged to map antagonistic interactions between MGEs. There are already several examples of this for CRISPR/Cas systems in MGEs, where the analyses of the spacers in the CRISPR array allowed the identification of the MGEs being targeted by the system [18,49,104].
- Fighting pathogenic bacteria. Virulence factors and antimicrobial resistance genes are frequently carried by MGEs. A better understanding of the defense, addiction, or attack systems that these elements employ to ensure their propagation might lead to the identification of novel strategies to counteract the spread of these costly elements, for instance, by favoring competing harmless MGEs. The presence of antiphage systems on MGEs could also promote the rapid evolution of resistance to phage therapies, and conversely, the identification of counter-defenses deployed by phages and other MGEs might provide solutions for the selection or engineering of more potent therapeutic phages.

Above all, to understand the roles of defense and/or counter-defense systems, given their abundance in MGEs, one must attain a better understanding of the complex networks of interactions between these semiautonomous agents and the host. Their study will shed novel light on the function, evolution, and ecology of microbes.

## Abbreviations

CRISPR/Cas: clustered regularly interspaced short palindromic repeats/CRISPR associated protein
HGT: horizontal gene transfer
ICE: integrative conjugative element
MGE: mobile genetic element
R–M: restriction–modification
